# Supplementary material for: Online conversion of reconstructed neural morphologies into standardized SWC format
Source: Nat Commun. 2023 Nov 16;14:7429. doi: 10.1038/s41467-023-42931-x (PMC10654402; doi:10.1038/s41467-023-42931-x)
Supplement: Supplementary file 1 — Supplementary Information [file 41467_2023_42931_MOESM1_ESM.pdf]

a

Metadata Descriptor Tag	: Value/Information
# CONTRIBUTOR	: Von Gersdorff
# REFERENCE	: doi.org/10.7554/eLife.81992
# CREATURE	: zebra finch
# SEX	: male
# AGE	: 120 days
# REGION	: Adjacent Dorsal Intermediate Arcopallium (AId)
# CLASS	: glutamatergic projection neuron
# CONDITION:	: control
# LABEL	: biocytin
# SLICING	: 180 micrometers coronal
# MICROSCOPY	: oil 60x
# COORDINATE	: micrometers
# ORIGINAL_SOURCE	: ShuTu

Index	Type	X	Y	Z	R	Parent
1	1	0.0	0.0	0.0	5.4	-1
2	3	12.9	-4.7	-3.5	1.3	1
3	3	14.4	-5.4	-3.1	0.8	2
4	3	16.0	-6.0	-3.5	0.8	3
⋮	⋮	⋮	⋮	⋮	⋮	⋮

b

Index	Type	X	Y	Z	R	Parent		
⋮	⋮	⋮	⋮	⋮	⋮	⋮		
3667	6	153.9	274.0	280.4	0.6	3666		
3668	6	151.0	274.4	280.3	0.5	5		
3669	6	150.6	274.4	279.9	0.3	3668		
3670	6	153.7	274.9	282.8	0.9	1		
connector_id	*x*	*y*	*z*	*treenode_id*	*prepost*	*label*	*partner*	*transmitter*
Unique ID	*Synapse cartesian coordinates*			*Index of node closest to synapse*	*0: input synapse 1: output synapse*	*Neurite structural domain*	*Unique ID for partnering neuron*	*Putative neurotransmitter*
# start synapse								
# 122753723	7248	17524	9798	3352	1	axon	880323584	gaba
# 117042798	14851	21367	5732	702	1	axon	851459584	gaba
# 128113988	6698	16653	12179	3031	1	primary.dendrite	850627072	gaba
# 175627002	18517	29104	28221	2340	1	dendrite	5901197312	gaba
⋮	⋮	⋮	⋮	⋮	⋮	⋮	⋮	⋮
# end synapse								

Supplementary Figure 1. Recommended optional inclusion of ancillary information in SWC Files. **a** Metadata information can be included as a header, **b** Synapse connectivity information can be included in the footer.

Supplementary Table 1. Mass validation for the most popular reconstruction formats on NeuroMorpho.Org

Legend		Count	
	Supported (SWC)		
	Supported (non-SWC)		
	NOT Supported (or Failed during Validation)		
	Number of SWC files	88898	
	Number of non-SWC files	143131	
	Total *(NeuroMorpho.Org Version 8.4.0)*	232029	

Software Name	Format	Count	Conversion/Standardization Success Rate
Aivia	swc	194	100.00%
Amira	am	488	73.33%
Amira	asc	4	100.00%
Amira	swc	1968	100.00%
Arbor	ntr	2	100.00%
Arbor	swc	97	100.00%
Catmaid	hoc	3	0.00%
Catmaid	ser	2	0.00%
Catmaid	swc	3986	100.00%
Custom (Amira)	am	16048	100.00%
Custom	asc	1	100.00%
Custom	hoc	9	100.00%
Custom (HOC)	nrn	4	100.00%
Custom (NTS)	out	62	93.55%
Custom	swc	7789	100.00%
Custom (Nevin NTS)	txt	54	96.30%
Custom (NeuroML)	xml	299	100.00%
Eutectic	asc	71	100.00%
Eutectic	geo	5	0.00%
Eutectic	swc	3	100.00%
Eutectic	nts	563	100.00%
Eyewire	swc	395	100.00%
Farsight	swc	2877	100.00%
GTree	swc	274	100.00%
Imaris	asc	10	100.00%
Imaris	dat	8	50.00%
Imaris	hoc	3049	99.86%
Imaris	ims	12020	99.81%
Imaris	imx	292	0.00%
Imaris	iv	6	0.00%
Imaris	swc	43604	100.00%
Imaris	zip	3	100.00%
Janelia Workstation-Large Volume Viewe	swc	1007	100.00%
Knossos	nml	3261	100.00%
Knossos	swc	1165	100.00%
Knossos	xml	837	100.00%
LinLab	swc	65	100.00%
NeuroGPS-Tree	swc	227	100.00%
Neurolucida	asc	19672	99.86%
Neurolucida	asc2	1	100%
Neurolucida	dat	59268	100.00%
Neurolucida	hoc	30	83.33%
Neurolucida	nrn	26	100.00%
Neurolucida	nrx	2810	100.00%
Neurolucida	swc	1180	100.00%
Neurolucida	traces	157	100.00%
Neurolucida	txt	12	0.00%
Neurolucida	xml	96	100.00%
Neuromantic, NeuronStudio	swc	20	100.00%
Neuromantic	asc	33	100.00%

-- Table T1 cont. --

Neuromantic	swc	2297	100.00%
NeuronJ	ndf	7844	100.00%
NeuronJ	swc	1249	100.00%
NeuronJ	txt	345	100.00%
NeuronStudio	eswc	118	100.00%
NeuronStudio	nst	3	0.00%
NeuronStudio	swc	3829	100.00%
Neuron_Morpho	swc	85	100.00%
Neurozoom	swc	80	100.00%
neuTu, Raveler	swc	1275	100.00%
neuTu	swc	1352	100.00%
neuTube	eswc	39	100.00%
neuTube	swc	3217	100.00%
nTracer	swc	53	100.00%
Orion	swc	10	100.00%
PyKNOSSOS	nmx	1021	100.00%
Raveler	swc	379	100.00%
Reconstruct	swc	17	100.00%
ShuTu	swc	14	100.00%
Simple Neurite Tracer	asc	61	100.00%
Simple Neurite Tracer	hoc	1	0.00%
Simple Neurite Tracer	ndf	24	100.00%
Simple Neurite Tracer	swc	5897	100.00%
Simple Neurite Tracer	traces	13146	100.00%
Simple Neurite Tracer	zip	69	100.00%
SparseTracer	swc	2	100.00%
Tablet	swc	14	100.00%
TRAKA	hoc	2	100.00%
TrakEm2	nml	7	0.00%
TrakEm2	swc	21	100.00%
TREES toolbox	mat	962	100.00%
Trees Toolbox	mtr	260	100.00%
Trees Toolbox	swc	260	100.00%
Vaa3D	eswc	22	100.00%
Vaa3D	swc	3944	100.00%
Viking	swc	52	100.00%
Viking	traces	1	100.00%

**Total no. of files**	**232029**

-- Table T1 End --
