# Supplementary material for: Online conversion of reconstructed neural morphologies into standardized SWC format
Source: Nat Commun. 2023 Nov 16;14:7429. doi: 10.1038/s41467-023-42931-x (PMC10654402; doi:10.1038/s41467-023-42931-x)
Supplement: Supplementary file 3 — Description of Additional Supplementary Files [file 41467_2023_42931_MOESM3_ESM.pdf]

Description of Additional Supplementary Files

File Name: Supplementary Data 1
Description: Example standard SWC file with metadata header.

File Name: Supplementary Data 2
Description: Example standard SWC file with synaptic connectivity information in footer

File Name: Supplementary Movie 1
Description: Overview of the SWC format specification.

File Name: Supplementary Movie 2
Description: Usage demonstration of the xyz2swc online file converter.
