# Supplementary material for: Online conversion of reconstructed neural morphologies into standardized SWC format
Source: Nat Commun. 2023 Nov 16;14:7429. doi: 10.1038/s41467-023-42931-x (PMC10654402; doi:10.1038/s41467-023-42931-x)
Supplement: Supplementary file 8 — Reporting Summary [file 41467_2023_42931_MOESM8_ESM.pdf]

nature portfolio

Corresponding author(s): Giorgio A. Ascoli

Last updated by author(s): Oct 10, 2023

# Reporting Summary

Nature Portfolio wishes to improve the reproducibility of the work that we publish. This form provides structure for consistency and transparency in reporting. For further information on Nature Portfolio policies, see our Editorial Policies and the Editorial Policy Checklist.

## Statistics

For all statistical analyses, confirm that the following items are present in the figure legend, table legend, main text, or Methods section.

| n/a | Confirmed | |
|---|---|---|
| ☐ | ☒ | The exact sample size ($n$) for each experimental group/condition, given as a discrete number and unit of measurement |
| ☒ | ☐ | A statement on whether measurements were taken from distinct samples or whether the same sample was measured repeatedly |
| ☒ | ☐ | The statistical test(s) used AND whether they are one- or two-sided<br>*Only common tests should be described solely by name; describe more complex techniques in the Methods section.* |
| ☒ | ☐ | A description of all covariates tested |
| ☒ | ☐ | A description of any assumptions or corrections, such as tests of normality and adjustment for multiple comparisons |
| ☒ | ☐ | A full description of the statistical parameters including central tendency (e.g. means) or other basic estimates (e.g. regression coefficient) AND variation (e.g. standard deviation) or associated estimates of uncertainty (e.g. confidence intervals) |
| ☒ | ☐ | For null hypothesis testing, the test statistic (e.g. $F$, $t$, $r$) with confidence intervals, effect sizes, degrees of freedom and $P$ value noted<br>*Give P values as exact values whenever suitable.* |
| ☒ | ☐ | For Bayesian analysis, information on the choice of priors and Markov chain Monte Carlo settings |
| ☒ | ☐ | For hierarchical and complex designs, identification of the appropriate level for tests and full reporting of outcomes |
| ☒ | ☐ | Estimates of effect sizes (e.g. Cohen's $d$, Pearson's $r$), indicating how they were calculated |

*Our web collection on statistics for biologists contains articles on many of the points above.*

## Software and code

Policy information about availability of computer code

| | |
|---|---|
| Data collection | All reconstruction data was sourced from NeuroMorpho.Org v8.4 which makes the original data files available for download as provided by the contributors in the format in which they were initially collected. |
| Data analysis | Custom code [DOI: 10.5281/zenodo.8429037] available at https://github.com/NeuroMorpho/xyz2swc/tree/main.<br>NeuronLand (g++-11, HDF5); TREES Toolbox, Bonfire (Octave 4.2.2); HBP Morphology Viewer (Node.js 18.15); SNT (FIJI 2020); natverse (R 4.0.0); skeletor (Python 3.8.5); Bootstrap v1.11.0. |

For manuscripts utilizing custom algorithms or software that are central to the research but not yet described in published literature, software must be made available to editors and reviewers. We strongly encourage code deposition in a community repository (e.g. GitHub). See the Nature Portfolio guidelines for submitting code & software for further information.

## Data

Policy information about availability of data

All manuscripts must include a data availability statement. This statement should provide the following information, where applicable:

- Accession codes, unique identifiers, or web links for publicly available datasets
- A description of any restrictions on data availability
- For clinical datasets or third party data, please ensure that the statement adheres to our policy

All digital reconstruction data used in this study is publicly available for download from the NeuroMorpho.Org (version 8.4) repository at https://neuromorpho.org.

The reconstruction files are made available in the format that they were collected-in and provided by the contributors, as well as their equivalent SWC representations. In addition for convenience, a small subset of the data sufficient to interpret and verify the functioning of the xyz2swc converter software is also deposited on GitHub: https://github.com/NeuroMorpho/xyz2swc/tree/main/input/to_convert.

## Human research participants

Policy information about studies involving human research participants and Sex and Gender in Research.

| | |
|---|---|
| Reporting on sex and gender | The study did not involve human research participants. |
| Population characteristics | The study did not involve human research participants. |
| Recruitment | The study did not involve human research participants. |
| Ethics oversight | The study did not involve human research participants. |

Note that full information on the approval of the study protocol must also be provided in the manuscript.

# Field-specific reporting

Please select the one below that is the best fit for your research. If you are not sure, read the appropriate sections before making your selection.

☒ Life sciences ☐ Behavioural & social sciences ☐ Ecological, evolutionary & environmental sciences

For a reference copy of the document with all sections, see nature.com/documents/nr-reporting-summary-flat.pdf

# Life sciences study design

All studies must disclose on these points even when the disclosure is negative.

| | |
|---|---|
| Sample size | We selected all 232,029 digital reconstruction files available in the NeuroMorpho.Org repository, to date the largest freely accessible database of neural reconstructions. |
| Data exclusions | No data was excluded from the analyses. |
| Replication | All experiments (individual file conversions) were performed at least twice, and the results were replicated successfully. |
| Randomization | It is not relevant to this study as we use the entire available dataset to assess conversion accuracy. |
| Blinding | It is not relevant to this study as we use the entire available dataset to assess conversion accuracy. |

# Reporting for specific materials, systems and methods

We require information from authors about some types of materials, experimental systems and methods used in many studies. Here, indicate whether each material, system or method listed is relevant to your study. If you are not sure if a list item applies to your research, read the appropriate section before selecting a response.

## Materials & experimental systems

| n/a | Involved in the study | |
|---|---|---|
| ☒ | ☐ | Antibodies |
| ☒ | ☐ | Eukaryotic cell lines |
| ☒ | ☐ | Palaeontology and archaeology |
| ☒ | ☐ | Animals and other organisms |
| ☒ | ☐ | Clinical data |
| ☒ | ☐ | Dual use research of concern |

## Methods

| n/a | Involved in the study | |
|---|---|---|
| ☒ | ☐ | ChIP-seq |
| ☒ | ☐ | Flow cytometry |
| ☒ | ☐ | MRI-based neuroimaging |
